# Imatinib induced severe skin reactions and neutropenia in a patient with gastrointestinal stromal tumor

**DOI:** 10.1186/1471-2407-10-438

**Published:** 2010-08-18

**Authors:** Jun-Eul Hwang, Ju-Young Yoon, Woo-Kyun Bae, Hyun-Jeong Shim, Sang-Hee Cho, Ik-Joo Chung

**Affiliations:** 1 Department of Hematology-Oncology, Chonnam National University Medical School, Gwangju 501-757, South Korea; 2 The Brain Korea 21 Project, Center for Biomedical Human Resources, Jeonnam Regional Cancer Center, Chonnam National University Hwasun Hospital, Hwasun, Jeollanam-do, South Korea

## Abstract

**Background:**

Imatinib mesylate has been used for the treatment of unresectable or metastatic gastrointestinal stromal tumors (GIST). The current recommended dose of imatinib is 400 mg/day that is increased to 800 mg/day in cases with disease progression. However, imatinib can be associated with diverse adverse events, which has limited its use. We report a case of severe adverse skin reactions with neutropenic fever during imatinib treatment in a patient with GIST.

**Case presentation:**

A 71-year-old man was admitted with a one month history of epigastric pain and a palpable mass in the right upper quadrant. An abdominal CT scan revealed a 20 × 19 cm intraabdominal mass with tumor invasion into the peritoneum. Needle biopsy was performed and the results showed spindle shaped tumor cells that were positive for c-KIT. The patient was diagnosed with unresectable GIST. Imatinib 400 mg/day was started. The patient tolerated the first eight weeks of treatment. However, about three months later, the patient developed a grade 4 febrile neutropenia and a grade 3 exfoliative skin rash. The patient recovered from this serious adverse events after discontinuation of imatinib with supportive care. However, the skin lesions recurred whenever the patient received imatinib over 100 mg/day. Therefore, imatinib 100 mg/day was maintained. Despite the low dose imatinib, follow up CT showed a marked partial response without grade 3 or 4 toxicities.

**Conclusion:**

The recommended dose of imatinib for the treatment of GIST is 400 mg/day but patients at risk for adverse drug reaction may benefit from lower doses. Individualized treatment is needed for such patients, and we may also try sunitinib as a alternative drug.

## Background

Imatinib mesylate is a selective tyrosine kinase inhibitor. It has become the gold standard treatment for unresectable or metastatic gastrointestinal stromal tumors (GIST). It has inhibitory activity against, BCR-ABL, c-KIT, and PDGFR [1,2]. The most common adverse events associated with imatinib include edema that is most frequently periorbital, nausea, diarrhea, muscle cramps, fatigue, skin rash, headache, and abdominal pain. Imatinib induced grade 3-4 neutropenia and skin rash may occur in as many as 7.1% and 3.8% of GIST patients, respectively [3,4]. The toxicities are generally mild or moderate and of grade 1 or 2 severity (NCI-CTC). Rarely, serious gastrointestinal or intraabdominal hemorrhage can occur in patients with large, bulky tumors [2,3]. Most cutaneous reactions are mild, however, severe reactions such as exfoliative dermatitis, toxic epidermal necrolysis and Stevens Johnson syndrome can also occur [5]. Sunitinib has demonstrated efficacy in treating patients with GIST who have experienced disease progression on or intolerance to imatinib [6]. However, due to the effectiveness of imatinib, imatinib may need to be used again in some patients despite a history of severe reactions. We report a case of severe adverse skin reactions with neutropenic fever during imatinib treatment.

## Case presentation

**Figure 1:**
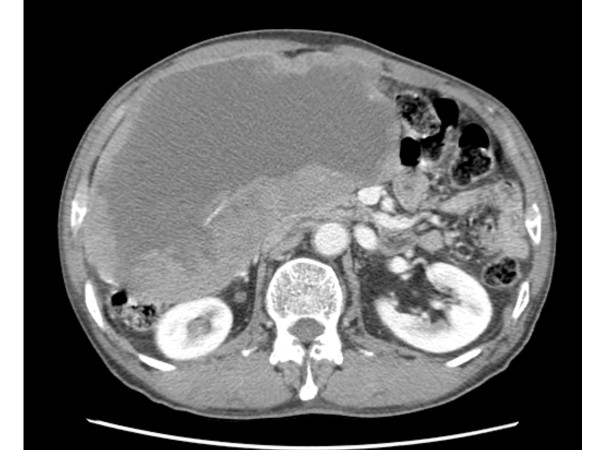
**Initial CT scan showed a 20 × 19 cm intraabdominal mass with central necrosis in the infra-hepatic space and tumor invasion into the peritoneum**.

**Figure 2:**
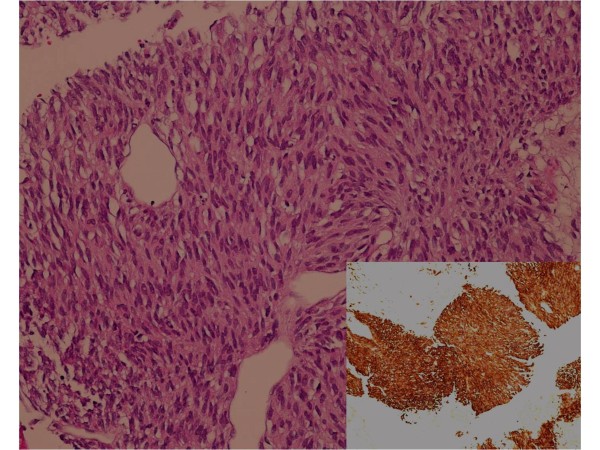
**Microscopic finding of the intraabdominal GIST demonstrating spindle cells (H&E; magnification, × 20)**. The tumor cells were strongly positive for c-KIT (small picture, magnification, × 10).

**Figure 3:**
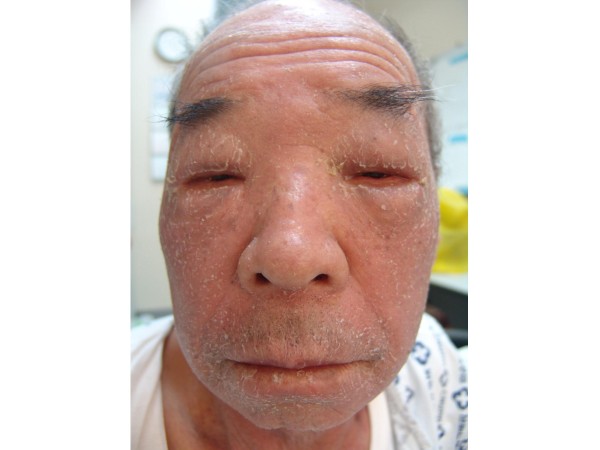
**The patient had marked periorbital edema, and an erythematous maculopapular rash covered by whitish dry scales affecting the face**.

A 71-year-old man was admitted with a one month history of epigastric pain and a palpable mass in the right upper quadrant. The patient had no significant past medical history. A CT scan of the abdomen showed a 20 × 19 cm intraabdominal mass with central necrosis in the infra-hepatic space and tumor invasion into the peritoneum (Figure 1). For a definitive diagnosis, ultrasound guided needle biopsy was performed. The results showed spindle shaped tumor cells that were strongly positive for c-KIT by CD 117 immunohistochemical staining; other immunohistochemical staining was negative including actin, desmin, S-100 and CD 34 (Figures 2). The mitotic figures were >5/50 high power field. Because the mass invaded the peritoneum, the patient was diagnosed with unresectable GIST. Given this diagnosis, treatment with imatinib at a dose of 400 mg daily was started. The patient tolerated the first eight weeks of treatment; the only toxicities were mild periorbital edema and pruritus. However, about three months after beginning treatment, the patient developed mild diarrhea, a grade 4 febrile neutropenia, and a grade 3 generalized erythematous maculopapular rash covered by whitish dry scales affecting the face, trunk and upper limbs (Figures 3). The patient did not developed any other lymphadenopathy and there were no oral mucosal lesions. The patient was admitted to the hospital and reported general weakness, loss of appetite, and visual problems including conjunctival suffusion and itching of both eyes. The periorbital edema was so severe that he could not open his eyes. The vital signs showed a high fever (temperature of 38.6°C, blood pressure 120/70 mmHg, heart rate 72/minute, and respiratory rate 18/minute). Laboratory examination revealed neutropenia, eosinophilila and a mild anemia, that is, white blood cell count 2.5 × 10^9^/L (reference value 4-10.8 × 10^9^/L), absolute neutrophil count 0.15 × 10^9^/L (reference value > 1.5 × 10^9^/L), eosinophil count 1.25 × 10^9^/L (percent eosinophil 50%) (reference value percent eosinophil 0-7%), hemoglobin 10.2 g/dl (12-18 g/dl), platelet count 218 × 10^9^/L (130-450 × 10^9^/L). Other blood chemistries revealed alkaline phosphatase 386 IU/L (39-117 IU/L), aspartate aminotransferase 66 IU/L (7-38 IU/L), alanine aminotransferase 161 IU/L (4-43 IU/L), lactate dehydrogenase 498 IU/L (218-472 IU/L), total bilirubin 3.9 mg/dL (0.35-1.3 mg/dL), blood urea nitrogen 29 mg/dL (8-23 mg/dL), creatinine 1.4 mg/dL (0.5-1.3 mg/dL). Mild hepatic and renal dysfunction was suspected. The patient was taking no other medication except for imatinib. Therefore, administration of imatinib was promptly discontinued, and a dermatological consultation was obtained.

The cutaneous findings were compatible with the diagnosis of an exfoliative dermatitis due to imatinib. The patient was managed with hydration and parenteral injections of a third generation cephalosporin as well as granulocyte colony stimulating factor. For the skin lesion management, systemic steroids (oral prednisone 30 mg/day) with topical steroid and emollient cream were provided. The patient recovered from the febrile neutropenia, and the eosinophil count normalized subsequently. The results of blood culture was negative and there were no infection foci. The skin lesions gradually improved with a reduction of the extension and severity of the erythema and scales. Further dermatological treatment was continued and one month later the rashes completely resolved with hyperpigmented areas remaining.

**Figure 4:**
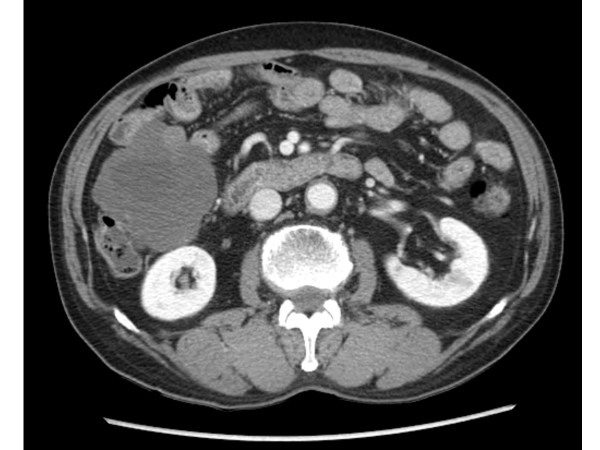
**Last follow up CT scan revealed a partial response**. The tumor decreased to 8 × 8 cm.

After the skin lesions improved, that is, one month later after stopping the imatinib, the drug was started at a dose of 100 mg every other day for 1 week without any complications. The dose was increased to 100 mg daily and the patient tolerated this dose. Subsequently the imatinib was increased to 200 mg daily, and then the periorbital edema and exfoliative skin lesions recurred on the forth day after the dose increase. Laboratory examination also showed increased liver enzyme, AST 89 IU/L, ALT 133 IU/L, and LDH 941 IU/L. We tried several times to increase the dose of imatinib. However, severe exfoliative dermatitis, periorbital edema, and elevation of liver enzymes developed each time. The combined steroid therapy was somewhat effective, but the exfoliative skin lesions progressed. After complete recovery, the imatinib was started again at a dose of 100 mg daily and this was maintained. Serial CT was performed to evaluate the tumor response. The first follow up CT was performed three months after starting the imatinib medication. The CT showed a partial response according to RECIST criteria, that is, the tumor decreased from 20 × 19 cm to 14 × 8 cm. At the second follow up CT after the patient received imatinib for about 3 weeks, during the prior 3 months due to severe hypersensivity reaction the CT showed a decrease in the tumor. Therefore, the patient has been maintained on imatinib 100 mg daily with CT follow up every 3-months. Despite the low dose imatinib, the last follow up CT revealed a prolonged partial response, and the tumor decreased to 8 × 8 cm (Figure 4).

## Conclusion

Gastrointestinal stromal tumors (GIST) are a subtype of sarcomas that have a unique natural history. Imatinib mesylate, a selective tyrosine kinase inhibitor, has become the gold standard for the treatment of unresectable or metastatic GIST [1].

Skin rashes are a well recognized side effect of imatinib treatment. In most cases, the lesions are mild, self limiting, and easily managed with anti-histamines or topical steroids, whereas a short course of oral steroids can be used to treat more severe cases [7]. Grade 3 or 4 skin rashes may occur in 3.8% of patients. In some patients, severe rashes can develop with desquamative components, including a report of Steven-Johnson syndrome [8-10]. In such cases, immediate discontinuation of therapy and systemic steroids are indicated. Severe skin lesions that were resistant to supportive measures have been the most frequent cause for permanent discontinuation of imatinib therapy. However, the frequency of this event is small (<1% of all patients) [7].

Standard management of drug induced skin rashes usually includes discontinuation of the suspected drug and avoidance of further exposure to this drug in the future. However, due to the effectiveness of imatinib, most oncologists try to maintain or reinitiate treatment with imatinib. To overcome severe skin reactions, several methods such as temporary discontinuation of imatinib treatment, once weekly dosing, a lower daily dose with or without a short course of an oral corticosteroid, and gradual dose escalation, have been reported [11,9,14].

In this case, febrile neutropenia resolved as soon as treatment was initiated and the skin rashes resolved gradually over one month. We started the imatinib again, but we could not increase the dose above 100 mg/day due to recurrence of the lesions. However, even with the low dose of imatinib, 100 mg/day, follow up CT demonstrated a decreased tumor mass. We could also try sunitinib which had demonstrated efficacy in treating patients with GIST who have experienced disease progression on or intolerance to imatinib [6]. However, the patient had good tumor response to imatinib, therefore we tried to maintain the imatinib.

Several studies have reported a dose related skin toxicity of imatinib, indicating a pharmacological effect of imatinib. This case can also mainly be related to the pharmacological effect of imatinib, but the delayed type hypersensitivity might be involved in some aspects like other skin rashes considering eosinophilia and pruritus [15-17]. The cause of the skin rashes is unclear. However, the platelet derived growth factor receptor is found abundantly in keratinocytes, and its inhibition by imatinib may play a role in the occurrence of this reaction [14]. Other investigators have proposed that certain skin reactions may result from an inhibition of KIT, found in basal cells [7,14,18]. However, it is unclear why these severe skin rashes develop in only a minority of patients.

Recently, Demetri et al. suggested that imatinib trough levels at steady state were associated with a clinical benefit [19]. According to this report, patients with an imatinib trough concentration, below 1,100 ng/ml, showed a shorter time to progression (11.3 months) and lower rate of clinical benefit. In our case, the trough concentration at steady state was 331 ng/ml. It remains to be seen whether the tumor will progress earlier or maintain a partial response. However, to date, the patient has showed a good tumor response over 14 months. The current recommended daily dose of imatinib is 400 mg, however, patients at risk for adverse drug reactions may benefit from lower doses. Individualized treatment is needed for such patients, and we may also try sunitinib as a alternative drug.

In conclusion, we describe a patient with an intraabdominal GIST that had a good tumor response, although we could not increase the dose of imatinib above 100 mg/day due to severe adverse skin reactions.

## Consent

Written informed consent was obtained from the patient for publication of this case report and any accompanying clinical images, including the image of the face.

## Competing interests

The authors declare that they have no competing interests.

## Authors' contributions

Jun-Eul Hwang is main author. YJY, WKB, HJS, SHC, IJC made substantial contributions to the conception and interpretation of clinical data and case related studies, and clinical decisions. All authors read and approved the final manuscript.

## Pre-publication history

The pre-publication history for this paper can be accessed here:

http://www.biomedcentral.com/1471-2407/10/438/prepub
